# Exploring the Efficacy of the Ilizarov Method for Infected Femoral Non-unions in Pediatric and Adult Populations

**DOI:** 10.7759/cureus.79357

**Published:** 2025-02-20

**Authors:** Muhammad Saqib, Muhammad Anwar Ullah, Saeed Ahmad, Muhammad Assad Javed, Tariq Ahmad, Rahim Khan

**Affiliations:** 1 Orthopedic Surgery, Gajju Khan Medical College, Swabi, PAK; 2 Neurosurgery, Timergara Teaching Hospital, Timergara, PAK; 3 Trauma and Orthopedics, Ghurki Trust Teaching Hospital, Lahore, PAK; 4 Orthopedics, Peshawar Medical College and Allied Hospital, Peshawar, PAK; 5 Neurosurgery, Akbar Niazi Teaching Hospital, Islamabad, PAK; 6 Orthopedic Surgery, Medical Teaching Institution (MTI) Mardan Medical Complex, Mardan, PAK

**Keywords:** adult, bone healing, complication rates, ilizarov method, infected femoral non-unions, pediatric

## Abstract

Background: Both pediatric and adult populations have considerable issues due to infected femoral non-unions, which may complicate healing and negatively influence quality of life.

Objective: This study aimed to evaluate the efficacy of the Ilizarov method in treating infected femoral non-unions in pediatric and adult populations, focusing on functional outcomes, complication rates, and infection control.

Methodology: A prospective observational study was conducted over one year, from January 2023 to December 2023. Patients who had the Ilizarov method and had been diagnosed with infected femoral non-unions ranged in age from five to 60. Demographic information, clinical presentations, surgical techniques, and postoperative results were gathered. IBM SPSS Statistics for Windows, Version 27.0 (Released 2020; IBM Corp., Armonk, New York, United States) was used to analyze the data. The results of pediatric and adult patients were compared using the t-test. P-values less than 0.05 were regarded as statistically significant.

Results: A total of 274 patients were included in the study, with 100 patients in the pediatric group (36.5%) and 174 patients in the adult group (63.5%). The mean age of the pediatric group was 12.60±3.20 years, while the mean age of the adult group was 36.50±12.90 years. According to the Association for the Study and Application of the Method of Ilizarov (ASAMI) criteria, excellent outcomes were observed in 60% (60 patients) of the pediatric group and 58.62% (102 patients) of the adult group. Radiological evaluations revealed that bone union was achieved in 80% (80 patients) of pediatric cases and 85.06% (148 patients) of adult cases. The mean time to bone union was shorter in the pediatric group, at 6.00±1.10 months, compared to 7.00±1.20 months in the adult group. Additionally, complications occurred in 15% (15 patients) of the pediatric group and 22.41% (39 patients) of the adult group.

Conclusion: The Ilizarov method is an effective treatment for infected femoral non-unions in both age groups, with pediatric patients exhibiting faster recovery and better outcomes.

## Introduction

Femoral non-unions pose serious problems for both pediatric and adult populations, especially when infection is involved [1]. In order to promote bone healing and restore function, the care of these patients is typically complicated and calls for the use of novel surgical procedures [2]. By hindering the body's natural healing processes and jeopardizing the efficacy of conventional treatment approaches, infection makes the situation worse [3]. Non-unions may result in long-term impairment and negatively affect quality of life since weight-bearing femur bones are essential for movement [4].

Because of the combination of poor local healing capability and persistent infection, conventional surgical methods, such as internal fixation and bone grafting, have shown little efficacy in treating infected femoral non-unions [5]. These procedures may also include the risk of further issues such as implant failure, infection recurrence, and prolonged hospital stays [6]. As a result, in order to handle these challenging situations, orthopedic surgeons are increasingly turning to alternate approaches like the Ilizarov procedure [7].

Because the Ilizarov approach may treat infection and bone abnormalities at the same time, it has become more and more popular as a distraction osteogenesis technique that uses an external fixator [8,9]. By means of regulated mechanical stimulation, the circular fixator device facilitates progressive bone regeneration while offering solid mechanical support [10]. It may also be continuously adjusted, which makes it very flexible in response to the changing needs of infection management and bone repair [11]. When previous therapies have failed to cure complicated instances of infected non-unions, this approach has been very helpful [12].

Although the Ilizarov procedure has demonstrated promising outcomes in both adult and pediatric patients, the overall effectiveness of this method in treating infected femoral non-unions remains a subject of debate. Notably, there is a scarcity of studies directly comparing its outcomes between pediatric and adult populations, limiting the generalizability of findings across different age groups.

Research objective

This study aims to assess the efficacy of the Ilizarov method in managing infected femoral non-unions in both pediatric and adult populations, with a specific focus on functional outcomes (evaluated using the Association for the Study and Application of the Method of Ilizarov (ASAMI) criteria), complication rates, and infection control through clinical and laboratory markers.

## Materials and methods

Study design and setting

This prospective observational study was conducted at the Medical Teaching Institution (MTI) Mardan Medical Complex, Mardan, Pakistan, from January 2023 to December 2023. The study focused on evaluating the effectiveness of the Ilizarov method in treating infected femoral non-unions in both pediatric and adult patients.

Inclusion and exclusion criteria

Patients were selected using a non-randomized consecutive sampling method, ensuring that all eligible cases meeting the inclusion criteria during the study period were enrolled. This approach minimized selection bias by avoiding arbitrary case inclusion while ensuring a representative sample of pediatric and adult patients with infected femoral non-unions. The inclusion criteria for this study encompassed patients aged 5-60 years who were diagnosed with infected femoral non-unions and had undergone the Ilizarov technique during the study period. The study population included both pediatric patients aged 5-17 years and adult patients aged 18-60 years, provided they had at least one year of follow-up after surgery. Patients who had non-infected femoral fractures, those with systemic infections or multiple bone diseases, and those who had received alternative treatments for infected non-unions were excluded from the study.

Sample size

To determine an appropriate sample size, the World Health Organization (WHO) formula for estimating proportions was utilized to ensure statistical robustness. Based on a 95% confidence level (Z=1.96), an anticipated success rate of 80% (p=0.80), and a 5% margin of error (E=0.05), the estimated sample size was 246 patients. To accommodate a 10% dropout rate, the final sample size was increased to 274 patients, ensuring the reliability and generalizability of the study findings.

Follow-up protocol

Each patient underwent structured follow-up assessments at one, three, six, and 12 months post-surgery. Clinical and laboratory markers were evaluated at each visit to monitor treatment effectiveness. Radiological evaluations were conducted to assess bone healing, while infection control was monitored using complete blood count (CBC), erythrocyte sedimentation rate (ESR), and C-reactive protein (CRP) levels. Functional outcomes were assessed based on the ASAMI criteria, and pain levels were measured using a visual analog scale (VAS). Additionally, postoperative complications, including pin tract infections and mechanical issues, were documented throughout the follow-up period.

Data collection

A standardized data collection form was used to ensure uniformity in recording patient information. This form included demographic details such as age, sex, and comorbidities, along with clinical presentation, infection history, and any prior treatments. Surgical details, including the duration of the procedure and any modifications to the Ilizarov technique, were also recorded. Postoperative outcomes were documented, focusing on bone healing, infection resolution, and any complications encountered. Functional recovery was assessed using the ASAMI criteria to provide an objective measure of patient progress.

Postoperative management

All patients followed a standardized postoperative protocol to optimize treatment outcomes. Infection control measures included an initial course of empirical intravenous antibiotics, followed by targeted therapy based on culture and sensitivity results. Pain management was administered through a stepwise analgesic regimen, starting with nonsteroidal anti-inflammatory drugs (NSAIDs) and escalating to opioids if required. Physical rehabilitation was initiated early, incorporating weight-bearing exercises and physical therapy to restore mobility and enhance functional recovery.

Statistical analysis

Statistical analysis was conducted using IBM SPSS Statistics for Windows, Version 27.0 (Released 2020; IBM Corp., Armonk, New York, United States). Descriptive statistics were used to summarize demographic and clinical characteristics. Comparisons between pediatric and adult patients were performed using independent t-tests for continuous variables such as time to bone union, while chi-squared tests were applied to categorical variables such as infection clearance rates. ANOVA (F-test) was employed to compare postoperative follow-up duration across different groups. To account for potential confounding factors affecting bone healing and infection control, demographic and clinical variables such as age, gender, comorbidities (diabetes, smoking history, and immunosuppressive conditions), infection chronicity, and prior treatments were systematically recorded. Multivariate analysis was performed to assess the independent effect of these factors on treatment outcomes. Additionally, subgroup analyses were conducted to compare outcomes within specific patient categories, ensuring a more accurate evaluation of the Ilizarov method's effectiveness across different clinical conditions. A p-value of less than 0.05 was considered statistically significant, ensuring the robustness of the findings.

Ethical approval

The Institutional Review Board (IRB) of Bacha Khan Medical College (BKMC), Mardan, granted the research ethical approval (approval number: 619/BKMC; date: 05/01/2023). Before being included in the study, all patients or their guardians provided written informed permission. Every step of the process complied with the Declaration of Helsinki's ethical guidelines.

## Results

The demographic and clinical characteristics of the pediatric (n=100) and adult (n=174) groups undergoing the Ilizarov method are summarized in Table 1. The pediatric group had a significantly lower mean age (12.60±3.20 years) compared to the adult group (36.50±12.90 years). Males constituted a slightly higher proportion in both groups (n=55 (55%) in pediatrics vs. n=105 (60.34%) in adults). Trauma was the primary cause of injury in both groups, with a higher incidence in pediatrics (n=75; 75%) than adults (n=115; 66.09%), whereas pathological injuries were more frequent in adults (n=59; 33.91%). Chronic infections were more prevalent than acute infections in both groups, with a higher percentage in adults (n=94; 54.02%). *Staphylococcus aureus* was identified in 30 (30%) of pediatric cases and 50 (28.74%) of adult cases. The Ilizarov technique was used for all patients, with the mean surgical duration being significantly longer in adults (124.20±28.70 minutes) than in pediatric patients (115.20±20.10 minutes; p=0.012).

**Table 1:** Demographic and clinical characteristics of pediatric and adult groups undergoing the Ilizarov method

Variable		Pediatric group (n=100)	Adult group (n=174)
Age in years	Mean±SD	12.60±3.20	36.50±12.90
Gender (n; %)	Male	55 (55%)	105 (60.34%)
	Female	45 (45%)	69 (39.66%)
Initial injury type (n; %)	Trauma	75 (75%)	115 (66.09%)
	Pathological	25 (25%)	59 (33.91%)
Infection type (n; %)	Acute	40 (40%)	80 (45.98%)
	Chronic	60 (60%)	94 (54.02%)
Microbial isolate (n; %)	Staphylococcus aureus	30 (30%)	50 (28.74%)
	Others	70 (70%)	124 (71.26%)
Surgical technique (n; %)	Ilizarov technique	100 (100%)	174 (100%)
Duration of surgery	Minutes	115.20±20.10	124.20±28.70

Table 2 presents the patient demographics and treatment outcomes. Significant differences were observed in several parameters between the pediatric and adult groups. The duration of non-union was significantly shorter in pediatric patients (8.50±2.90 months) compared to adults (10.10±3.60 months; p<0.001; t=3.54). Similarly, pediatric patients achieved bone union faster (6.00±1.10 months vs. 7.00±1.20 months; p<0.001; t=3.52). Treatment duration was also significantly shorter in the pediatric group (90.57±15.29 days) than in adults (95.34±18.42 days; p=0.006; t=2.75). However, the postoperative follow-up duration was identical in both groups (12.00±1.52 months in pediatrics and 12.00±2.01 months in adults; p=1.000; F=1.15), indicating no significant difference in follow-up periods.

**Table 2:** Patient demographics and treatment outcomes

Outcome variable	Pediatric group (n=100)	Adult group (n=174)	P-value	Statistical value
Age (years)	12.60±3.20	36.50±12.90	<0.001	t=23.45
Duration of non-union (months)	8.50±2.90	10.10±3.60	<0.001	t=3.54
Duration of surgery (minutes)	115.20±20.10	124.20±28.70	0.012	t=2.53
Time to bone union (months)	6.00±1.10	7.00±1.20	<0.001	t=3.52
Treatment duration (days)	90.57±15.29	95.34±18.42	0.006	t=2.75
Postoperative follow-up duration (months)	12.00±1.52	12.00±2.01	1.000	F=1.15

Table 3 highlights the clinical and radiological outcomes in both groups. The ASAMI criteria showed comparable results, with the majority achieving excellent or good outcomes (n=60 (60%) excellent and n=30 (30%) good in pediatric patients vs. n=102 (58.62%) excellent and n=54 (31.03%) good in adults; p=0.812; χ²=0.058). Bone union was achieved in 80 (80%) of pediatric cases and 148 (85.06%) of adult cases, with no significant difference between the groups (p=0.342; χ²=0.902). Infection resolution was high in both groups (n=90 (90%) in pediatrics vs. n=160 (91.95%) in adults; p=0.623; χ²=0.241). Similarly, laboratory markers such as CRP level reduction (n=85 (85%) vs. n=155 (89.08%); p=0.412; χ²=0.673) and white blood cell (WBC) count normalization (n=75 (75%) vs. n=140 (80.46%); p=0.356; χ²=0.855) showed no significant differences. Complication rates were slightly higher in adults (n=39; 22.41%) compared to pediatric patients (n=15; 15%), but the difference was not statistically significant (p=0.148; χ²=2.09).

**Table 3:** Clinical and radiological outcomes ASAMI: Association for the Study and Application of the Method of Ilizarov; CRP: C-reactive protein; WBC: white blood cell

Outcome variable		Pediatric group (n=100)	Adult group (n=174)	P-value	Statistical value
ASAMI criteria (n; %)	Excellent	60 (60%)	102 (58.62%)	0.812	χ²=0.058
	Good	30 (30%)	54 (31.03%)		
	Fair	8 (8%)	16 (9.16%)		
	Poor	2 (2%)	2 (1.15%)		
Radiological evaluation (n; %)	Bone union achieved	80 (80%)	148 (85.06%)	0.342	χ²=0.902
	Non-union	20 (20%)	26 (14.94%)		
Infection control assessment (n; %)	Resolution of infection (yes)	90 (90%)	160 (91.95%)	0.623	χ²=0.241
	Resolution of infection (no)	10 (10%)	14 (8.05%)		
Clinical and laboratory markers (n; %)	CRP levels decreased	85 (85%)	155 (89.08%)	0.412	χ²=0.673
	WBC count normalized	75 (75%)	140 (80.46%)	0.356	χ²=0.855
Complication rates (n; %)	Complications present	15 (15%)	39 (22.41%)	0.148	χ²=2.09
	No complications	85 (85%)	135 (77.59%)		

## Discussion

The Ilizarov method has garnered significant interest as an effective treatment for infected femoral non-unions, particularly due to its potential for achieving bone union and infection control. In our study, the pediatric cohort (n=100) had a mean age of 12.60±3.20 years, whereas the adult group (n=174) had a mean age of 36.50±12.90 years. The relatively narrow age distribution among pediatric patients aligns with existing literature, which suggests that children present with distinct injury patterns and healing responses compared to adults [13]. These age-related differences may influence healing dynamics and surgical outcomes when using the Ilizarov technique.

Our findings showed that 60% of pediatric patients and 58.62% of adult patients achieved good functional outcomes based on the ASAMI criteria. The bone union rate was 80% in pediatric patients and 85.06% in adults, indicating comparable effectiveness of the Ilizarov method across age groups. These results are consistent with prior research, which suggests that children may benefit from their higher regenerative potential and greater capacity to adapt to mechanical stressors [14,15]. Additionally, the mean time to bone union was significantly shorter in pediatric patients (6.00±1.10 months) than in adults (7.00±1.20 months), supporting the notion that younger patients have a biological advantage in bone healing and remodeling [16].

The infection resolution rate was 90% in pediatric patients and 91.95% in adults, reinforcing the Ilizarov method's role in managing complex infections associated with non-unions. These findings align with previous studies reporting similar infection clearance rates [17]. Clinical markers such as CRP levels improved in 85% of pediatric patients and 89.08% of adults, further demonstrating a parallel trend in inflammatory response reduction across both age groups.

In terms of complications, 15% of pediatric patients and 22.41% of adults experienced post-treatment complications. This trend aligns with prior studies indicating lower complication rates in younger patients, likely due to their superior biological adaptability and faster healing capacity [14,18]. Our findings support the Ilizarov method as a viable treatment for infected femoral non-unions across age groups, though controlled studies are needed to confirm its superiority over other treatments. However, it is important to note that our study did not include a control group or comparison with alternative treatment modalities, limiting the ability to attribute these outcomes solely to the Ilizarov technique. While we accounted for some clinical variables, potential confounding factors such as comorbidities, smoking, and nutritional status may have influenced treatment outcomes. Future studies incorporating randomized controlled designs and larger sample sizes are necessary to further validate these findings.

Strengths and limitations

This study has several strengths, particularly in its prospective observational design, which allows for the systematic tracking of clinical outcomes over time. The inclusion and exclusion criteria were clearly defined to minimize selection ambiguity, ensuring a well-characterized study population. Additionally, the sample size calculation followed standard statistical methods, incorporating a 10% dropout adjustment to maintain statistical power. To ensure the reliability of outcome assessment, the study employed validated measures such as the ASAMI criteria, which are widely accepted for evaluating functional recovery in limb reconstruction. The use of structured follow-up intervals (one, three, six, and 12 months) further strengthened data consistency and minimized variability in outcome assessment.

To reduce potential bias, we implemented several measures. Selection bias was minimized by applying standardized inclusion and exclusion criteria, ensuring that only eligible patients with infected femoral non-unions were included. Additionally, all data collection followed a uniform protocol, where demographic details, clinical presentation, surgical factors, and postoperative outcomes were recorded using a standardized form to maintain consistency. Observer bias was reduced by involving multiple independent evaluators in assessing clinical, radiological, and functional outcomes, ensuring that subjective assessments were not influenced by individual perspectives. Moreover, statistical analysis accounted for variations by using adjusted comparisons between pediatric and adult subgroups, and appropriate statistical tests (t-tests, chi-squared tests, ANOVA) were applied to control for variability in continuous and categorical variables.

Despite these efforts, the study has some limitations, particularly the lack of a control group, which limits direct comparisons with alternative treatment modalities. Additionally, while efforts were made to control confounding variables through subgroup analysis, factors such as patient age, comorbidities (diabetes, smoking, immunosuppressive conditions), and previous treatments were not explicitly controlled through randomization. The one-year follow-up period also may not fully capture long-term complications or functional recovery, necessitating extended monitoring in future studies. Given these constraints, the findings should be interpreted as correlative rather than causal. Future research should focus on multi-center studies with larger cohorts and randomized controlled trials (RCTs) to provide stronger evidence for the Ilizarov technique's effectiveness in managing infected femoral non-unions.

## Conclusions

Our research indicates that the Ilizarov technique is a feasible and successful therapy for infected femoral non-unions in adults and children alike. The results show that pediatric patients had improved recovery dynamics, quick bone repair, and good functional outcomes together with efficient infection management. These findings highlight the need to take age into account when developing treatment plans for infected non-unions, as younger patients may respond more favorably to healing. All things considered, the Ilizarov approach seems to be a useful choice for managing the intricate problems related to infected femoral non-unions, eventually leading to better patient outcomes for patients of all ages.
